# ASO Author Reflections: Preoperative Radiation Boost Reduces Re-Excision Rate and Locoregional Treatment Time in Early-Stage Breast Cancer Patients

**DOI:** 10.1245/s10434-025-18271-z

**Published:** 2025-09-02

**Authors:** Molly A. Chakraborty, Bruce G. Haffty

**Affiliations:** 1 Rutgers New Jersey Medical School, Newark, NJ USA; 2 Department of Radiation Oncology, Rutgers Cancer Institute of New Jersey, New Brunswick, NJ USA

## Past

A radiation tumor bed boost for breast cancer patients is typically delivered after breast-conserving surgery (BCS), either integrated with or sequentially after whole-breast radiation therapy (WBRT). This is based on convention and the limited historic data demonstrating poor wound healing after preoperative radiation.^1,2^ However, these studies focused on WBRT and not a radiation boost, which may offer advantages such as reduced locoregional treatment time due to operating room scheduling limitations and downsizing of the tumor prior to BCS, thus possibly resulting in lower rates of re-excision after initial lumpectomy. In the initial results of this phase II clinical trial, in which patients with early-stage breast cancer received a preoperative radiation boost, we showed that the rates of wound complications were non-inferior to the standard rates for patients receiving a postoperative boost.^3^ In this study, we aimed to show that the rate of re-excision after initial lumpectomy was lower, and the time from diagnostic biopsy to completion of radiation therapy was shorter, for patients in this trial receiving a preoperative radiation boost compared with patients at our institution receiving a standard postoperative radiation boost.

## Present

We firstly showed that the rate of re-excision after initial lumpectomy in this trial, which was 3.4%, was significantly lower than the literature-reported rate of 17.2% (*p* = 0.0005) and was also lower than the rate in a contemporary cohort of patients at our institution receiving the Canadian hypofractionation regimen with sequential boost if indicated (13.48%; *p* = 0.015).^4^ This is important as a lower re-excision rate could lead to a reduction in treatment costs, improved patient experience, better cosmetic results, reduced rates of surgical complications, and earlier initiation of systemic therapies. We then showed that the median locoregional treatment time was 109 days (range 42–258) in this trial, which was significantly shorter than the median treatment time in the contemporary cohort receiving the Canadian hypofractionation regimen (126 days, range 74–278; *p* < 0.0001) and the median treatment time for patients at our institution in a previous clinical trial with the same fractionation scheme as the current trial, but with a sequential boost (122 days, range 62–311; *p* = 0.0002).^4^ Although the differences in locoregional treatment time may not be clinically significant, it was very appreciated by the patients in the trial.

## Future

As this was a single-institution study with a relatively small sample size, investigating re-excision rates and locoregional treatment time in a larger, multi-institutional, randomized trial would further support these results. A randomized, multi-institutional trial to investigate preoperative radiation boost in patients undergoing oncoplastic procedures is currently under consideration.
